# Bilateral interstitial keratitis in a patient with presumed brain tuberculoma

**DOI:** 10.5935/0004-2749.20230015

**Published:** 2022-01-31

**Authors:** Elaine Fiod Costa, João Victor Pimentel Oliveira, Luciano Moreira Pinto, Conceição de Maria Pedrozo e Silva Azevedo, Ana Luisa Hofling-Lima

**Affiliations:** 1 Departamento de Medicina I, Universidade Federal do Maranhão, São Luis, MA, Brazil.; 2 Universidade Federal do Maranhão, São Luis, MA, Brazil.; 3 Departamento de Oftalmologia, Escola Paulista de Medicina, Universidade Federal de São Paulo, São Paulo, SP, Brazil.; 4 Department of Ophthalmology and Visual Sciences, Escola Paulista de Medicina, Universidade Federal de São Paulo, São Paulo, SP, Brazil.

**Keywords:** Keratitis, Scleritis, Intracranial tuberculoma, Ocular tuberculosis, Anemia, iron deficiency, Humans, Case report, Ceratite, Esclerite, Tuberculoma intracraniano, Tuberculose ocular, Anemia ferropriva, Humanos, Relato de caso

## Abstract

Interstitial keratitis is an inflammation of the corneal stroma without epithelium or endothelium involvement. The underlying causes are mostly infectious or immune mediated. Brazil has one of the highest incidence rates of tuberculosis in the world. Tuberculosis is considered one of the causes of interstitial keratitis. Malnutrition and anemia are risk factors of the disseminated disease. This is a case report of a 10-year-old child who presented with decreased visual acuity and a clinical diagnosis of bilateral interstitial keratitis and sclero-uveitis. The patient had been treated with topical steroids with partial improvement. Examinations revealed severe iron deficiency anemia, negative serologies for human immunodeficiency virus and syphilis, positivity for cytomegalovirus- and herpes simplex-specific IgG, and purified protein derivative of 17 mm. During the follow-up, the patient presented with tonic-clonic seizures, and magnetic resonance imaging findings suggested a central nervous system tuberculoma. Interstitial keratitis improvement was observed after specific tuberculosis treatment. This is the first case report describing the association of interstitial keratitis and central nervous system tuberculoma.

## Abstract (pt)

A ceratite intersticial é uma inflamação do estroma corneano sem envolvimento epitelial ou endotelial causada principalmente por doenças infecciosas e imunomediadas. O Brasil tem altas taxas de tuberculose que deve ser lembrada como causa de ceratite intersticial. Desnutrição e anemia são fatores de risco da forma disseminada da tuberculose. Este é um relato de uma criança de 10 anos com redução de acuidade visual e diagnóstico clínico de ceratite intersticial bilateral e esclerouveíte. O paciente obteve melhora parcial da ceratite com corticoide tópico. Exames laboratoriais mostraram anemia ferropriva grave, sorologias negativas para HIV e sífilis; IgM negativo e IgG positivo para citomegalovírus e herpes simplex e PPD positivo (17 mm). Ele evoluiu com crises tônico-clônicas e a ressonância nuclear magnética revelou tuberculoma do sistema nervoso central. A melhora da ceratite intersticial foi observada após tratamento para tuberculose. Este é o primeiro caso que descreve a associação de ceratite intersticial e tuberculoma do sistema nervoso central.

## INTRODUCTION

Tuberculosis (TB), caused mainly by bacillus *Mycobacterium tuberculosis*, is considered a worldwide public health problem owing to its high prevalence and global incidence rates. TB is transmitted through aerosols that when inhaled, allow contact between bacilli and alveoli. In the first onset of tuberculous infection, the immune system keeps the infectious bacilli under control^(1)^. In 5% of cases, primary infection is not contained, resulting in primary TB^(1)^. Immunodeficiency disorders are risk factors for TB development. Such conditions include human immunodeficiency virus (HIV)/acquired immunodeficiency syndrome, organ transplantation, age < 5 years or >60 years, malnutrition, alcoholism, diabetes mellitus, and autoimmune diseases^(1)^.

The disseminated disease is represented by primary miliary TB, in which an extrapulmonary disease may occur^(1)^. The common sites of occurrence of extrapulmonary tuberculosis are the lymph nodes, pleura, and osteoarticular system. Central nervous system (CNS) TB is the most severe form of the disease and can present as meningitis, intracranial tuberculomas, and abscesses. Tuberculomas are granulomas formed by an interaction between the mycobacterial pathogen and the host’s immune response and may cause hydrocephalus and other symptoms indicative of a CNS mass lesion^(2)^.

Interstitial keratitis (IK) manifests as peripheral corneal inflammation with anterior stromal infiltrates, usually associated with anterior uveitis and stromal neovascularization. More frequently, it occurs secondary to an immunologic response and may be attributed mostly to infectious diseases such as Lyme disease, Epstein-Barr virus infection, congenital or acquired syphilis, herpes simplex, herpes zoster, or TB^(3)^. We report the case of a 10-year-old child diagnosed as having tuberculoma who experienced IK as the first symptom of the disease. Owing to the high incidence of tuberculosis in Brazil, this diagnosis should be part of the laboratory investigation and always put in mind.

## CASE REPORT

This is a case report of a 10-year-old child from São Luis, Maranhão, Brazil. The patient’s first complaint was eye redness and reduced visual acuity. Over a 6-month period, the boy underwent several treatments with topical corticosteroids, with partial improvement of the symptoms. The patient had no previous disease or treatment, underwent the schedule vaccination in accordance with the Brazilian guidelines, and presented with a bacillus Calmette-Guérin (BCG) vaccination scar.

**Figure 1:**
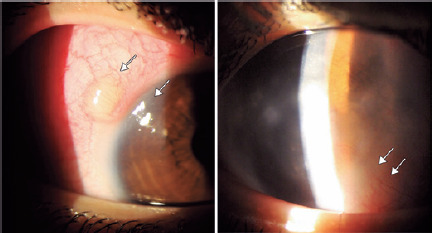
Biomicroscopy photographs of the right eye, presenting with temporal-superior nodular scleritis and deep peripheral stromal corneal opacity with interstitial vascularization (left). The left eye shows denser deep stromal diffuse cornea opacity in the periphery with interstitial corneal neovascularization (right).

In the ophthalmic examination, the best-corrected visual acuity (BCVA) was 20/25 in the right eye (oculus dexter [OD]) and counting fingers in the left eye (oculus sinister [OS]). Slit-lamp examination of the OD revealed conjunctival hyperemia 2+/4+, peripheral corneal opacity with deep neovascularization, and mild anterior chamber reaction 1+/4+. The OS had conjunctival hyperemia 1+/4+ and diffuse corneal opacity with evident deep neovascularization of the cornea suggestive of IK associated with sclero-uveitis (Figure 1). Fundoscopy revealed no abnormalities in the OD but was impossible in the OS owing to corneal opacity. The ocular ultrasonography finding was normal in the OS.

Laboratory examination revealed a blood count indicating severe iron deficiency anemia (hemoglobin level, 8.57 g/dl; mean corpuscular volume, 69.1 fL) and leukocytosis (10,200 cells/µl; normal range, 4,500-8,500 cells/µl), with normal neutrophil, monocyte, eosinophil, and typical lymphocyte counts. The serologies for HIV and syphilis were negative; the IgM and IgG specific to cytomegalovirus and herpes simplex were negative and positive, respectively; and the PPD test result was positive (17 mm).

**Figure 2:**
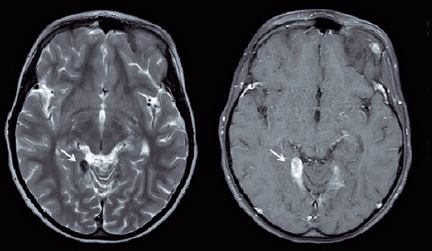
Bilateral nodular thickening of the cerebellar tentorium, with homogeneous and intense enhancement after the use of intravenous contrast media, more evident on the right side.

Two weeks after the initial examination, the child presented tonic-clonic seizures, disorientation, and confused speech. On physical examination, he was eupneic, afebrile, hydrated, and had no lymph node enlargement. Neurological examination revealed no meningeal or focal signs. Seizure treatment was started with phenobarbital administration. Electroencephalography revealed no abnormalities. Head computed tomography revealed findings suggestive of calcification. Cerebrospinal fluid analysis revealed normal pressure and appearance, with normal chloride and protein levels, low glucose level (50 mg/dl; normal range, 60-80 mg/dl), and high lactic dehydrogenase level (36 U/l; normal range, <24 U/l). From, the global and specific cytology analyses, the following values were obtained: leukocytes, 4/mm^3^ and erythrocytes, 11/mm^3^. Head magnetic resonance imaging (MRI) revealed bilateral meningeal nodular thickening with homogeneous enhancements in the cerebellar tentorium and axial T2, of approximately 2.6 × 1.0 cm, with features of CNS tuberculoma (Figure 2).

**Figure 3:**
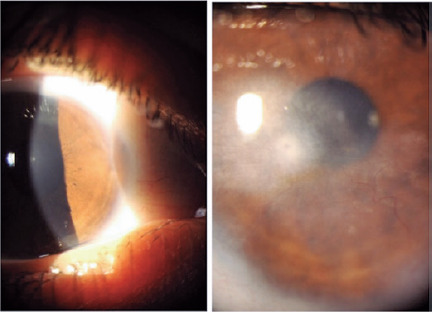
Biomicroscopy photographs after 6 months of treatment, showing improvement of interstitial keratitis in both eyes, but more evident in the right eye (left). The left eye (right) still presents a residual corneal opacity with peripheral deep stromal vascularization.

A 10-month tuberculosis treatment with isoniazid, rifampicin, pyrazinamide, and ethambutol for 2 months was initiated, and then isoniazid and rifampicin were administered for another 8 months. Within 2 months, topical corticosteroids were additionally administered for 45 days. After 6 months, the deep neovascularization of the cornea showed regression (Figure 3), and the BCVA was 20/20 in the OD and 20/60 in the OS.

## DISCUSSION

Ocular involvement is considered an uncommon manifestation of extrapulmonary TB and usually shows no correlation with or clinical evidence of lung disease. Ocular lesions can be caused by a direct invasion of microorganisms (active infection) or, more frequently, by immunologic reactions (delayed hypersensitivity type IV) in the absence of an infectious agent. The most frequent feature is posterior uveitis, and symptoms first appear from 10 to 14 weeks after bacillus inhalation and may persist for years if not treated^(4)^.

Only a few cases of IK related to TB have been reported. Three of these cases had ocular involvements other than keratitis, including anterior granulomatous uveitis^(5)^, sclerokeratitis and hypopyon^(6)^, and bilateral corneal perforation^(7)^. Only one case was related with another extrapulmonary manifestation, an osteoarti cular involvement^(8)^.

In children, CNS tuberculoma usually presents with increased intracranial tension, seizures, and localizing neurological signs. The disease may also present with have prolonged low-grade fever with or without vague behavioral disturbances or even remain silent for many months. Childhood tuberculomas can occur in the brain (parenchymal) but often originate from meninges or even ependyma (meningeal), adjacent to the dense basal meningovascular enhancement. MRI is the modality of choice for the assessment of potential tuberculomas, which show solid caseous necrosis centrally^(9)^.

Consensus has been reached in the literature about the protection provided by the BCG vaccine against severe and disseminated forms of TB in children. In the first 5 years, it has a protective efficacy against tuberculous meningitis and miliary TB of up to 86%; however, its efficacy rate may decrease to 59% at 10 and 15 years after vaccination^(10)^.

PPD response gauges the reaction to tuberculin. In general, a response of >10 mm is considered positive. In populations vaccinated with BCG, PPD response may be difficult to interpret. In general, indurations >15 mm are more related to infection with TB bacillus than to skin hypersensitivity caused by the vaccine^(10)^. In this case, the tuberculoma diagnosis was presumed on the basis of the PPD reading and MRI features.

Brazil has one of the highest incidence rates of TB in the world^(10)^. TB should be considered as a cause of IK. It is related to several individual and social factors such as immunodeficiency conditions, low income, housing conditions, and malnutrition. This is the first case report to describe the association of IK and CNS tuberculoma in a child. Malnutrition and high social vulnerability might have contributed to the disseminated disease. Iron deficiency can negatively affect cellular immunity, even before the child becomes anemic, and this can lead to an increase in susceptibility to illnesses.
